# 树突状细胞的体外培养及其抗癌作用

**DOI:** 10.3779/j.issn.1009-3419.2010.05.19

**Published:** 2010-05-20

**Authors:** 昌云 马, 芳 吴, 繁义 孔, 文艳 周

**Affiliations:** 061001 沧州，沧州市中心医院胸外科 Department of Toracic Surgery, Central Hospital of Cangzhou, Cangzhou 061001, China

**Keywords:** 树突状细胞, 肿瘤可溶性抗原, 金黄色葡萄球菌肠毒素A, 肺肿瘤, 超抗原, Dendritic cell, Tumor soluble antigen, Staphylococcal enterotoxin A, Lung neoplasms, Superantigen

## Abstract

**背景与目的:**

恶性肿瘤患者免疫力低下，缺乏强有力的抗肿瘤免疫应答，其原因是肿瘤细胞的抗原性较弱，而且抗原提呈细胞的功能低下，使肿瘤抗原不能有效地提呈给淋巴细胞。因此如何能有效地诱导出抗肿瘤免疫效应是一个非常关键的问题。本研究通过建立体外培养树突状细胞（dendritic cells, DC）的方法并观察其诱导的抗肺癌作用的研究，为以DC为基础的肺癌免疫瘤苗的临床应用提供实验依据。

**方法:**

3 mol/L氯化钾法提取人肺腺癌细胞GLC-82的可溶性抗原多肽；从人外周血单个核细胞（peripheral blood mononuclear cell, PBMC）中用GMCSF、IL-4和TNF-α体外诱导扩增DC，用流式细胞仪FCM及免疫染色法分析鉴定DC；肺癌肿瘤可溶性抗原（tumor soluble antigen, TSA）和超抗原金黄色葡萄球菌肠毒素A（staphylococcal enterotoxin A, SEA）联合修饰致敏DC；以联合抗原修饰后的DC、单纯肺癌抗原TSA致敏的DC、单纯超抗原SEA修饰的DC和未经抗原修饰的DC分别与外周血T淋巴细胞共同孵育，刺激T淋巴细胞活化增殖，诱导产生具有识别肺癌抗原的特异性CTL（作为效应细胞分别称为TSA-SEA-DCL、TSA-DCL、SEA-DCL、DCL）；采用MT法检测各效应细胞对靶细胞GLC-82、肺癌CALU-6和人红白血病K562细胞的抗癌活性；镜下观察DC、效应细胞形态及对靶细胞的杀伤过程。

**结果:**

诱导出高表达CD1a、HLADR、CD80具有典型树突状细胞特性的DC；TSA-SEA-DCL对靶细胞GLC-82的杀伤率明显高于TSA-DCL、SEA-DCL及DCL，亦明显高于对K562细胞的杀伤率；与靶细胞共育时镜下发现效应细胞CTL靠近并聚集在肿瘤细胞周围，致使肿瘤细胞发生变性坏死及凋亡。

**结论:**

本实验方法能成功诱导出具有典型特征的成熟DC，肺癌TSA联合超抗原SEA诱导的DC疫苗对肺癌细胞有高效特异杀伤作用。

## Abstract (en)

**Background and objective:**

Immunocompromised patients with malignant tumor always lack of strong anti-tumor immune response, because the antigenicity of tumor cells is weak, and antigen-presenting cell function is low, so that can not be effectively presenting tumor antigens to the lymphocytes. Terefore, how to effectively induce anti-tumor immune response is the key issue. Trough the study on establishing a method to culture dendritic cells (DC) *in vitro* and to observe the anti-lung cancer immunological effect induced by DC, we provided defnite experiment basis for the clinic application of vaccine based on DC.

**Methods:**

Trough the experiment we get the soluble antigen polypeptide from lung cancer cells GLC-82 by 3 mol/L potassium chloride. DCs are cultured and obtained from peripheral blood mononuclear cell by GM-CSF, IL-4 and TNF-a. DCs are identifed by flow cytometer (FCM) and immunostaining. DCs modifed by lung cancer tumor soluble antigen (TSA) and staphylococcal enterotox in A (SEA), DCs modifed by TSA or DCs modifed by SEA or DCs modifed by nothing were cultivated together with T lymphocyte, and the obtained cells are named TSA-SEA-DCL or TSA-DCL or SEA-DCL or DCL as effector cells. The anti-tumor activity of every effector cells against target cells was assayed with MT method. Shape of DCs and effector cells, and the process of killing target cells were observed in microscope.

**Results:**

Induced DCs expressed more CD1a, CD80 and HLA-DR, which had typical cell traits such as tree branch. The killing ratio of the TSA-SEA-DCL *in vitro* to GLC-82 is larger than TSA-DCL, SEA-DCL and DCL, also larger than to K562. When the effector cells cultivate with target cells, we can observe the CTL approach and gather to the cancer cell, induce it necrosis and apoptosis.

**Conclusion:**

Ripe DCs that have typical characteristic and phenotype could be induced successfully. High potency and relatively specifc antilung caner effect can be prepared in virtue of DC Bacterin Induced by lung caner TSA and SEA.

以往通过直接输入效应细胞或与免疫佐剂联合输入至体内以效应细胞形式用于抗肿瘤免疫治疗，由于存在抗原在体内不能被有效提呈或单纯的效应细胞在体内不能大量增殖等缺陷，所以它们的应用均受到限制。因而改变和促进免疫系统对肿瘤抗原的提呈以诱导有效的抗瘤免疫效应，成为肿瘤免疫治疗中具有实际价值的研究方向。树突状细胞（dendritic cells, DC）是人体内最有效的抗原递呈细胞（antigen presenting cell, APC），金黄色葡萄球菌肠毒素A（staphylococcal enterotoxin A, SEA）是一种外源性超抗原（superantigen, SAg），具有高效刺激T细胞增殖、增强免疫功能等生物学活性。我们从外周血单个核细胞中联合应用细胞因子诱导获得较高纯度的成熟DC，采用功能最强的APC即DC作为瘤苗载体，以肺癌肿瘤可溶性抗原（tumor soluble antigen, TSA）及超抗原SEA体外冲击致敏DC并诱导活化T淋巴细胞，诱导产生了相对高效而特异的抗癌免疫效应。为临床应用研究提供实验依据。

## 1 材料与方法

### 1.1 材料

人肺腺癌细胞株GLC-82、人红白血病细胞株K562（中国科学院肿瘤研究所）。hGM-CSF、hIL-4、hIL-2、hTNF-α（美国PEPRO TECH公司），鼠抗人CD3-FITC、CD8- FIT、CD14-FITC、CD1a-FITC、HLA-DR、CD80-FITC（北京中山公司），羊抗鼠IgG-FITC（Santa Cruz Bio-Tech公司），S-100蛋白染色试剂盒（SABC法，美国ZYMED公司），RPMI-1640、胎牛血清（FBS，GIBCO公司），胰蛋白酶、淋巴细胞分离液（Sigma公司），考马斯亮兰R250（Fluka公司），四甲基偶氮唑蓝（MTT，Sigma公司），二甲基亚砜（DMSO，Amresso公司），超抗原SEA（北京邦定公司）。

### 1.2 方法

#### 1.2.1 肺癌可溶性抗原提取

用3 mol/L KCl法提取人肺腺癌GLC-82细胞可溶性抗原：取5×10^8^个人肺腺癌GLC-82细胞，PBS溶液洗涤，1 000 r/min离心15 min，5倍体积的3 mol/L KCl 4 ℃震荡过夜，液体透析，12 000 r/min离心30 min，取上清。考马斯亮兰法测定可溶性抗原蛋白多肽含量。

#### 1.2.2 DC的体外培养扩增与鉴定

取健康成人外周抗凝全血50 mL，用淋巴细胞分离液密度梯度离心，常规获取单个核细胞（peripheral blood mononuclear cell, PBMC），调细胞浓度为2×10^6^/mL，加入24孔板置于5%CO_2_、37 ℃孵箱中培养2 h，洗除非贴壁细胞，每孔加入终浓度为100 ng/mL的GM-CSF、50 ng/mL的IL-4及含10%FBS的RPMI-1640完全培养基1 mL，培养扩增，于第5天加入终浓度为100 ng/mL的TNF-α，第7天收集DC细胞。光学、倒置相差显微镜及电镜形态学观察，SABC免疫细胞化学染色法观察DC细胞形态。用FCM分析检测DC表型。

#### 1.2.3 肺癌TSA和超抗原SEA修饰致敏DC

于DC培养的第5天，将制备的人肺腺癌GLC-82细胞可溶性抗原以终浓度50 ng/mL和超抗原SEA以终浓度50 μg/L加入实验组DC中，以此建立负载肺癌TSA和超抗原SEA的DC，同时设有未加SEA的TSA-DC对照组、未加TSA的SEA-DC对照组和未加TSA、SEA的DC对照组。

#### 1.2.4 T淋巴细胞的分离培养与诱导活化

用尼龙棉柱法分离获得T淋巴细胞：按前述方法获得的非贴壁PBMC调细胞浓度为2×10^6^/mL；将该细胞悬液加在尼龙棉柱上，孵箱培养30 min；RPMI-1640培养基洗脱尼龙棉柱，收集细胞悬液为T淋巴细胞。经FCM鉴定T细胞表型。以上得到的各组致敏DC按DC:T淋巴细胞分别为1:10的比例混合培养。倒置显微镜动态观察细胞形态、大小、数量等变化。至第7天收集活化的T淋巴细胞即CTL。各组作为效应细胞分别称为TSA-SEA-DCL、TSA-DCL、SEA-DCL、DCL。

#### 1.2.5 靶细胞杀伤试验

以上各组作为效应细胞，以人肺腺癌细胞GLC-82、人红白血病细胞K562作为靶细胞，效靶比按50:1共同培养12 h，用MTT显色法测定实验孔和对照孔570 nm处光密度（oplical Density, OD）值并计算杀伤率。杀伤率=[单独靶细胞OD值+单独效应细胞OD值-实验孔OD值]/单独靶细胞OD值×100%。镜下观察效应细胞对靶细胞的杀伤过程。

### 1.3 统计学处理

统计方法采用SPSS 10. 0版软件行*t*检验和方差分析，实验数据用Mean±SD表示，以*P* < 0.05为有统计学差异。

## 2 结果

### 2.1 DC形态观察及表型鉴定结果

PBMC培养2 h后细胞呈贴壁生长（图 1A），在DC培养第24 h后，可见贴壁的单个核细胞形成较均匀散布的聚集体，有少量细胞呈半悬浮，细胞体积较小，多为圆形；至第4天，半悬浮细胞明显增多，细胞大量增殖聚集成团，细胞体积较前增大，胞核呈不规则形态。少数细胞表面可见有毛刺状突起；待加入TNF-α与肺癌TSA、超抗原SEA后，于培养6天-7天，细胞数目增多体积增大，细胞逐渐从聚集变为分散悬浮状态，用相差显微镜可看到细胞形态不规则，可呈星状、树突状，细胞表面从饱满、透亮变为暗淡、边缘模糊，细胞表面大量毛刺状突起，呈现典型的DC形态（图 1B）。DC经流式细胞仪分析鉴定结果显示，用此方法诱导培养7天可获得大量高表达CD1a（65.2%）、HLADR（73.7%）、CD80（61.5%）低表达CD14（9.2%）的成熟型DC。SABC法免疫组化染色观察DC结果显示：阴性对照组细胞无染色，实验组仅阳性细胞胞浆内有弥漫分布的棕黄色颗粒，细胞形态不规则，有伸展的突起（图 1C）。透射电镜观察DC结果显示：DC有许多长短不一的蔓状突起和褶皱，构成DC不规则的外形。胞质内含有较多滑面内质网、糖原颗粒和线粒体，还可见空泡等，溶酶体及吞噬体少。细胞核常偏居一侧，核大而不规则，染色质丰富（图 1D）。

**1:**
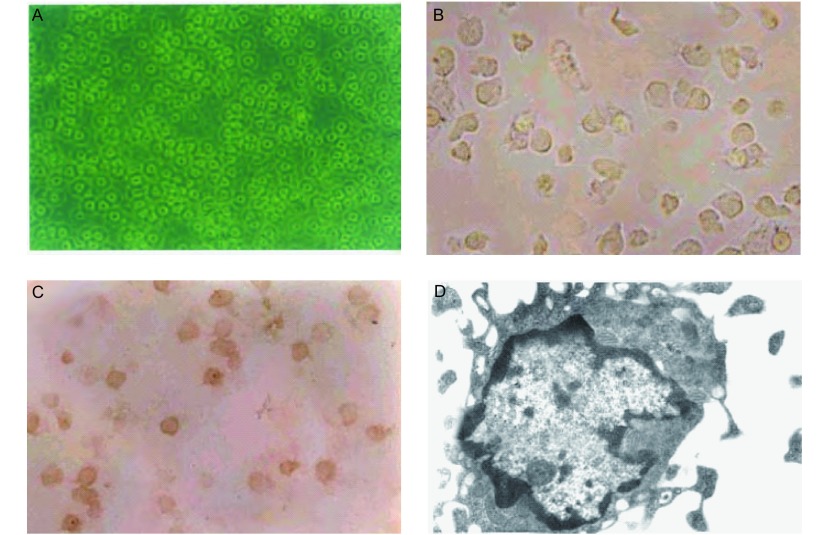
培养的DC细胞形态变化。A：培养2 h后贴壁生长的PBMC（即DC的前体细胞）（倒置相差显微镜，×200）；B：培养第7天的DC：细胞形态不规则，可见突起，胞体大，胞质颗粒增加（倒置相差显微镜，×400）；C：S-100蛋白免疫染色，示培养第7天的DC细胞有棕黄色颗粒的阳性染色（光学显微镜，×400）；D：电镜下的DC：细胞有许多长短不一的突起，核大而不规则，染色质丰富，胞质内有较多线粒体（透射电镜，×8 000）。 Morphological changes of cultured DC. A: After 2 h cultured adherent growth of PBMC (*ie* DC precursor cells) (inverted phase contrast microscope, ×200); B: 7-day cultured DC: irregular cell morphology can be seen protruding cell body, large increase in cytoplasmic granules (Inverted phase contrast microscope, ×400); C: S-100 protein immunostaining, showing the first seven days of the DC cultured cells stained positive brown particles (Optical microscope, ×400); D: Electron micrograph of DC: cells have many different lengths protruding nucleus rather than the rules of chromatin rich in cytoplasm, more mitochondria (TEM, ×8 000).

### 2.2 各组效应细胞形态观察结果

在DC与T淋巴细胞混合培养即联合修饰后的DC活化T淋巴细胞诱导CTL过程中发现：在混合培养48 h，DC刺激T细胞大量增殖；在混合培养第7天，T细胞围绕DC聚集成簇，诱导出特异性CTL细胞并增殖。

### 2.3 各种效应细胞对不同靶细胞的杀伤活性

用MTT法测得各种效应细胞对不同靶细胞的杀伤率，结果见表 1。由表可看出，经肺癌TSA、超抗原SEA联合修饰后的TSASEA-DCL组效应细胞对靶细胞GLC-82的杀伤活性明显高于未经联合抗原修饰的DCL组（*P* < 0.01）、未经肺癌可溶性抗原TSA致敏的SEA-DCL组（*P* < 0.01）和未经超抗原SEA修饰的TSA-DCL组（*P* < 0.05）；TSA-SEA-DCL对GLC-82的杀伤率明显高于对人红白血病细胞K562的杀伤率（*P* < 0.01），这说明TSA-SEA-DCL具有高效而特异性的杀伤作用；而SEA-DCL组和DCL组对不同靶细胞的杀伤率之间无统计学差异（*P* > 0.05），说明未经肿瘤抗原修饰的SEA-DCL和DCL无选择性杀伤靶细胞的表现。

**1:** 效应细胞对靶细胞的杀伤率比较（%） Effector cells killing of target cells compare to the rates (%)

Effector cells	GLC-82	K562
TSA-SEA-DCL	92.51±4.46^*^	41.94±2.12^**^
TSA-DCL	71.38±3.17^#^	31.66±1.65
SEA-DCL	33.64±2.15^△^	32.95±2.53^△△^
DCL	19.64±0.88^☆^	21.07±0.80^☆☆^
Compared with ^☆^, ^*^P < 0.01; Compared with ^△^, ^*^P < 0.01; Compared with ^#^, ^*^P < 0.05; Compared with ^**^, ^*^P < 0.01; Compared with ^△△^, ^△^*P*>0.05; Compared with ^☆☆^, ^☆^*P*>0.05.		

### 2.4 靶细胞杀伤实验的镜下观察

在TSA-SEA-DCL特异性杀伤靶细胞过程中，混合培养2 h，发现CTL很快靠近肿瘤细胞，并逐渐聚集在其周围及表面，共育4 h-6 h可见肿瘤细胞体积缩小，胞膜皱缩，细胞间隙变窄，贴壁程度下降，由贴壁逐渐回缩脱壁变圆形，漂浮于培养基中，少量肿瘤细发生变性坏死。电镜下可见与CTL共育4 h的GLC-82细胞出现胞质凝缩，核糖体、线粒体等聚集，核染色质浓缩、边集成新月状，核膜凹陷、被分割，继之可见核固缩、核膜出芽、染色质脱落，形成凋亡小体，细胞发生调亡（图 2）。共育12 h，死亡细胞数量增多，细胞形态多呈圆形或不规则形，有的似发芽状，有的伸出伪足样不规则突起，胞核明显固缩，染色质凝结成块状并边集，有些瘤细胞肿胀出现空泡，胞核肿胀，胞膜破裂，部分瘤细胞已经溶解，有些只剩下轮廓；小部分贴壁生长的细胞胞质颗粒增多，形态不规则，伸展性欠佳。共育24 h可见大量溶解的细胞碎片。而对照组GLC-82细胞贴壁生长较良好，K562为圆形悬浮细胞，充分生长。

**2:**
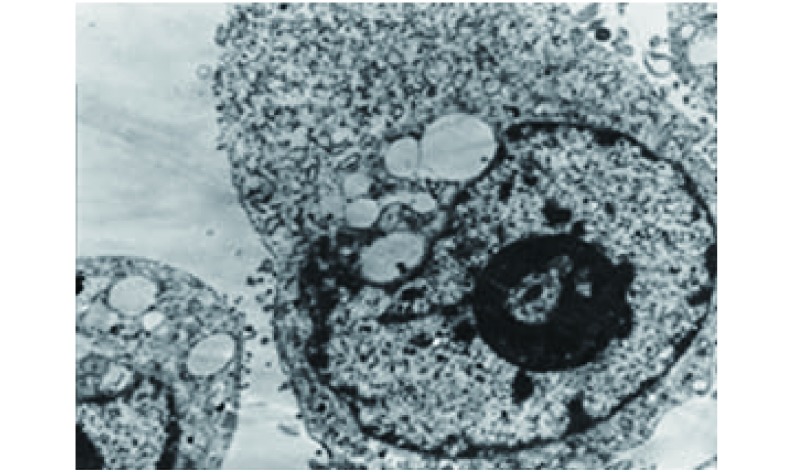
与诱导的CTL共育4 h的GLC-82细胞：胞质凝缩，核染色质浓缩、边集，核膜凹陷，细胞发生凋亡（透射电镜，×4 000） GLC-82 cells cultivated together with the induced CTL for 4 h: cytoplasmic condensation, nuclear chromatin condensation and margination, nuclear membrane depression, the cell apoptosis (TEM, × 4 000)

## 3 讨论

树突状细胞是目前所知的体内最强的抗原递呈细胞，从分化程度和成熟状态来说可分为成熟DC（mature DC, mDC）和未成熟DC（immature DC, imDC）^[1]^。未成熟的DC存在于全身非淋巴组织中，具有很强的摄取处理抗原的能力，一旦发育为成熟的DC, 捕获抗原的能力即丧失，成为能激活静息T细胞产生初次免疫应答的抗原递呈细胞。细胞因子是调节DC分化成熟的重要因素，不同细胞因子对DC的分化成熟存在不同影响。研究^[2, 3]^发现，在DC培养基中加入GM-CSF、IL-4，仅可诱导出iDC，这类DC能通过吞噬、吞饮作用或受体介导有效地摄取抗原，但刺激初始型T细胞的能力很弱，若给以一定的刺激信号如TNF-α、LPS、IL-1β、CD40L、肿瘤抗原、单核细胞条件培养基等，这些不成熟DC则进一步分化成熟，具备较强的抗原提呈能力及刺激初始型T细胞增殖的功能。有实验^[4]^证实，TNF-α等虽可以促使DC分化成熟，但其可显著下调DC胞吞能力及可溶性抗原摄取能力。故在DC负载抗原之前应用TNF-α不利于DC摄取抗原。本研究在借鉴前人培养DC的基础上，从外周血中获取PBMC，经贴壁培养得到粘附的单核细胞，再加GM-CSF和IL-4诱生出大量DC，在相差显微镜和电子显微镜下具有典型DC特征，较高地表达DC比较特征性的分子标志CD1a和蛋白S-100，高表达MHC-Ⅱ类分子HLA-DR及部分表达共刺激分子CD80等，而单核细胞表面标志CD14^+^的表达则随培养时间延长逐渐降低。有实验表明，DC在摄取抗原之后48 h内如果未遇到炎性因子或细胞因子的刺激，抗原就会在细胞内彻底降解，从而无法呈递于细胞表面，而且如果仅仅应用未成熟DC往往只能导致机体的免疫耐受，说明体外应用有效的促成熟因子至关重要。实验于DC负载抗原（DC培养的第4天）后24 h（即DC培养的第5天）加入具有促成熟及维持DC生长作用的促成熟因子TNF-α，有效地使DC上调表达协同刺激分子CD80，表达高水平多肽-MHC复合物，且能分泌IL-12等，具有强烈激发初始型T细胞增殖的能力，呈现成熟DC的特点。DC摄取抗原并将其处理成具有免疫原性的多肽，以MHC——抗原肽复合物的形式表达于其表面的同时，其膜表面的协同刺激分子与T细胞表面相应配体结合，进而激活抗原特异性T细胞，促其活化增殖，产生免疫应答^[5]^。

超抗原是高效的T细胞丝裂原，具有刺激T细胞增殖、增强免疫功能等生物学活性，它以完整的蛋白质分子形式直接与抗原呈递细胞膜的MHC-Ⅱ类分子抗原结合槽结合并且提呈给T细胞^[6]^。超抗原主要是通过以下2种途径发挥其对肿瘤细胞的杀伤作用。①SAg依赖的细胞介导的细胞毒作用：SAg激活的CD8^+^的杀伤力很强的抑制T细胞或CTL和CD4+的辅助性T细胞，直接或间接地杀伤表达有MHC-Ⅱ类分子的肿瘤细胞；②细胞因子参与的抑瘤作用：被激活的T细胞可释放多种细胞因子，非特异性地直接或间接杀伤肿瘤细胞，这些细胞因子主要包括肿瘤TNF-α、IL-2、IFN-γ等^[7]^。

单纯的抗原不能活化T淋巴细胞，必须经过APC递呈，因为T淋巴细胞活化需要抗原和辅助分子双信号才能有效地诱导CTL形成，发挥抗瘤效应。而肿瘤细胞可通过下调肿瘤相关抗原、低表达或不表达MHC分子和共刺激分子，使抗原不能被有效递呈给T细胞以产生特异性的抗瘤效应，因此激发有效的T细胞介导的抗瘤免疫反应是提高肿瘤免疫治疗效果的关键。负载肿瘤抗原的DC疫苗由于可通过DC表面MHC类分子、共刺激分子将各种已知或未知的肿瘤相关抗原信息得到有效递呈，因此作为理想的肿瘤疫苗在实验和临床研究受到广泛重视^[8, 9]^。肺癌细胞蛋白提取物中的肿瘤可溶性抗原刺激DC可能有多种不同的肿瘤特异抗原刺激DC，从而诱导针对不同抗原决定簇的CTL克隆等^[10]^。我们采用功能最强的抗原递呈细胞即DC作为瘤苗载体，以肺癌TSA及超抗原SEA体外冲击致敏DC并诱导活化T淋巴细胞，可以保证肿瘤抗原被有效摄取提呈，且可提供所需的共刺激信号，激发效应细胞大量增殖，诱导产生了相对高效且特异的抗癌免疫效应。
